# Supplementary figures and images for: Habitat association in the critically endangered Mangshan pit viper (Protobothrops mangshanensis), a species endemic to China
Source: PeerJ. 2020 Jul 1;8:e9439. doi: 10.7717/peerj.9439 (PMC7334975; doi:10.7717/peerj.9439)

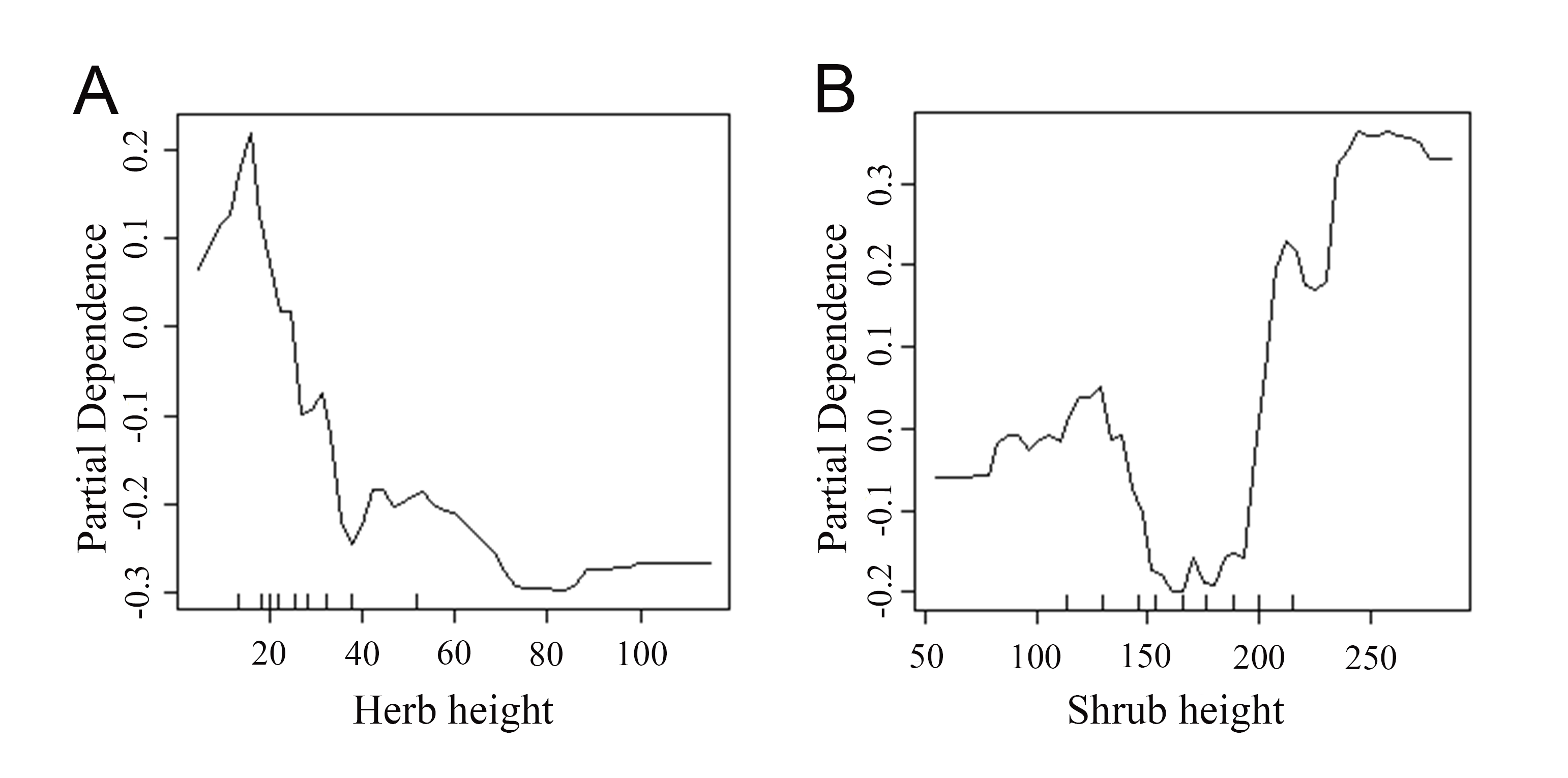

Supplement: Supplemental Information 1 — Partial dependance is the dependance of the probability of occurrence on one predictor variable after averaging out the effects of the other predictor variables in the model. [file peerj-08-9439-s001.png]
